# High SINE RNA Expression Correlates with Post-Transcriptional Downregulation of *BRCA1*

**DOI:** 10.3390/genes4020226

**Published:** 2013-04-29

**Authors:** Maureen Peterson, Vicki L. Chandler, Giovanni Bosco

**Affiliations:** 1 Department of Genetics, Geisel School of Medicine, Dartmouth College, Hanover New Hampshire, USA; E-Mail: Maureen.Peterson@dartmouth.edu; 2 Bio5 Institute, University of Arizona, Tucson Arizona, USA; E-Mail: Vicki.chandler@moore.org; 3 Department of Plant Sciences, University of Arizona, Tucson Arizona, USA

**Keywords:** SINE, retrotransposon, *BRCA1*, post-transcriptional, gene regulation

## Abstract

Short Interspersed Nuclear Elements (SINEs) are non-autonomous retrotransposons that comprise a large fraction of the human genome. SINEs are demethylated in human disease, but whether SINEs become transcriptionally induced and how the resulting transcripts may affect the expression of protein coding genes is unknown. Here, we show that downregulation of the mRNA of the tumor suppressor gene *BRCA1* is associated with increased transcription of SINEs and production of sense and antisense SINE small RNAs. We find that *BRCA1* mRNA is post-transcriptionally down-regulated in a *Dicer* and *Drosha* dependent manner and that expression of a SINE inverted repeat with sequence identity to a *BRCA1* intron is sufficient for downregulation of *BRCA1* mRNA. These observations suggest that transcriptional activation of SINEs could contribute to a novel mechanism of RNA mediated post-transcriptional silencing of human genes.

## 1. Introduction

Transposable elements have been shown to affect gene regulation in plants and fungi [1] and in some animal species [2]. How transposable elements may regulate specific genes in humans is not well understood, despite the fact that transposable elements constitute approximately half of the human genome [3]. Retrotransposons that replicate by an RNA intermediate are of particular interest since their RNAs can be directly targeted by a variety of post-transcriptional silencing mechanisms (reviewed in [4]). Retrotransposons known to be currently active in the human genome are long interspersed nuclear elements (LINEs), autonomous elements encoding their own enzymes needed for reverse transcription and genomic integration, and short interspersed nuclear elements (SINEs), non-autonomous elements that rely on the enzymes encoded within LINE sequences [5]. Recent experiments have implicated retrotransposons in establishing DNA methylation patterns in cell differentiation, in mammalian brain development, and in cancer [6,7,8,9,10,11]. In mouse, the Agouti *viable yellow* allele of the agouti gene contains a mouse specific retrotransposon that induces differential DNA methylation of retrotransposon DNA and results in differential expression of the agouti gene that determines coat color [12]. In human bladder cancers, overexpression of a transcript variant of the *MET* gene is driven by a LINE-1 promoter as the LINE-1 sequence becomes hypomethylated in cancer progression [13]. A current, complete review of the role transposable elements may play in cancers has recently been published [11]. Other mechanisms of retrotransposon *cis-*effects include the findings that *Alu* elements, primate specific SINEs, can play roles in alternative splicing, RNA editing, translational initiation, and transcription initiation and elongation [14,15,16].

**Figure 1:**
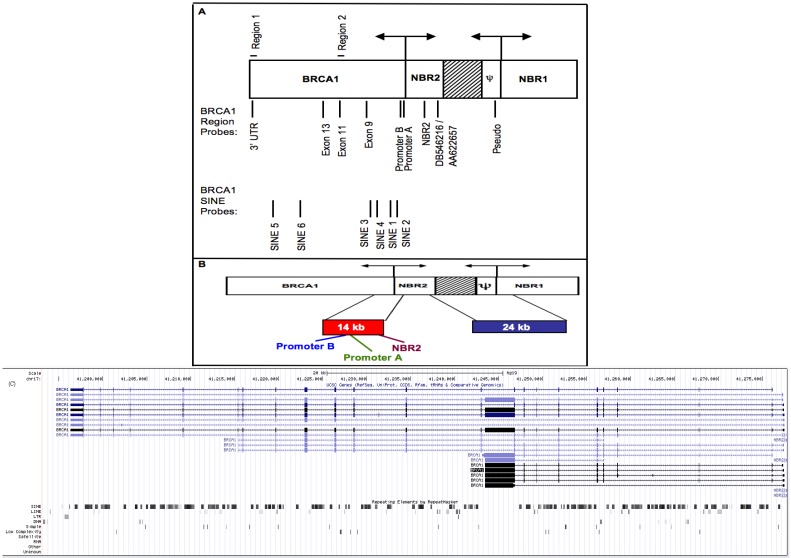
Human BRCA1 genomic structure. (A) The human *BRCA1* genomic region contains four genes and various types of repetitive sequences. The various regions presented are not to scale. A large intrachromosomal segmental duplication (shown in B) resulted in a partial pseudogene with homology to *BRCA1* (denoted as ψ) and a partial copy of *NBR1* (*NBR2*). Two small (<250 bp) promoters drive divergent transcription of the four genes. Hatched region: intergenic sequence. Arrows: direction of transcription from divergent promoters. Location of probes used in nuclear run-on and small RNA blot analyses are indicated, as are region 1 and 2 used in qRT-PCR experiments. (B) Location of two intrachromosomal segmental duplications, with lengths of 14 kb and 24 kb, in relation to *BRCA1* and how run-on probes in Figure 2 fall within them. The 14 kb duplication is represented by the red block and the 24 kb duplication is represented by the blue block. (C) Image from the UCSC genome browser of the *BRCA1* genomic locus. The location of SINEs and other repetitive sequences within *BRCA1* are indicated.

Many human genes contain the highly repetitive SINE sequences within their introns and untranslated regions, which results in transcripts containing SINE sequences. For example, the *BRCA1* locus contains over 500 SINEs embedded within its introns and one SINE within its 3’ UTR. Because it is comprised of repetitive sequences whose transcription is driven by small divergent promoters, the *BRCA1* genomic structure (Figure 1A) has the potential to generate transcripts from both strands. For example, transcription of *NBR2* across the *BRCA1* pseudogene sequence (denoted as ψ) would result in a transcript with homology to the functional *BRCA1* gene in the antisense orientation, thus raising the potential for double-stranded RNA and subsequent siRNA formation with homology to *BRCA1*. Additionally, *BRCA1* and has a high density of SINEs (Figure 1C), raising the possibility that *BRCA1* could be subject to transposable element mediated gene regulation within human cells. Given the abundance and sequence similarity among different SINEs, production of small RNAs from SINE elements located anywhere in the genome could target a variety of protein coding transcripts that have regions of SINE sequence homology. Whether *BRCA1* may be post-transcriptionally silenced is of particular interest because *BRCA1* is frequently downregulated in sporadic breast cancers without associated mutations in the coding or promoter regions [17,18]. Additionally, promoter hypermethylation occurs in only a small subset of the majority of sporadic breast cancers that exhibit *BRCA1* downregulation [19]. Thus, additional mechanisms of *BRCA1* downregulation remain to be discovered. Here, we explored the hypothesis that SINE sequences with homology to those found within the *BRCA1* locus function to post-transcriptionally downregulate *BRCA1* mRNA in human cultured cells. We found that *BRCA1* downregulation in multiple breast cancer cell lines was not associated with promoter methylation or a reduced transcription rate, but instead correlated with increased bidirectional SINE transcription and the presence of SINE and *BRCA1* small RNAs in cells where *BRCA1* was downregulated. Moreover, *BRCA1* mRNA downregulation could be rescued by depleting *Dicer* and *Drosha*, and in non-cancer cells partial reduction in *BRCA1* transcript levels could be achieved with ectopic transcription of double stranded SINE RNA with sequence identity to the *BRCA1* transcript. From these results, we suggest a model in which high levels of SINE RNAs contribute to post-transcriptionally downregulate *BRCA1* in human cells.

## 2. Experimental Section

### 2.1. Cell Culture

WI-38 VA-13 cells were provided by the lab of Kathleen Dixon and were originally established from fetal lung fibroblast [20]. HMEC were obtained from the University of Arizona Cancer Center stock collection and were established from reduction mammoplasty [21]. MCF10A were obtained from the University of Arizona Cancer Center stock collection and were established from fibrocystic disease tissue [22]. MDA-MB-231, MCF7, UACC2087, UACC893, and ZR-75-1 were obtained from the stock collection at the University of Arizona Cancer Center. MDA-MB-231, MCF7, and UACC2087 were established from pleural effusions [23,24,25] and UACC893 and ZR-75-1 were established from primary ductal carcinoma [26,27]. All lines were cultivated in the ATCC recommended media supplemented with 1 U/milliliter penicillin, 1 U/milliliter streptomycin, and 0.5 nanogram/liter amphotericin.

### 2.2. RNA Extraction, Reverse Transcription, and qRT-PCR

RNA was extracted using RNeasy Kit (Qiagen), treated with DNAse (Ambion), and subsequently purified using the RNA Clean and Concentrator Kit (Zymo). 1 milligram of total RNA was reverse transcribed using the i-Script cDNA synthesis kit (Bio-Rad). 1 milliliter of cDNA was used in a qPCR reaction. Data was quantified using MyIQ Software (Bio-Rad). qPCR reactions were carried out with an annealing temperature of 58 C using 300 nanomolar final primer concentration and an extension time of 45 seconds.

### 2.3. Nuclear Run-on

RNA probes were synthesized by *in vitro* transcription of PCR fragments carrying T3 promoter tails using T3 RNA Polymerase (Promega). Nuclei were extracted from approximately 75 million cells as described previously [28], with the exception that nuclei were directly isolated from human cultured human cells rather than ground tissue. Briefly, cells were trypsinized and pelleted, then washed once in ice cold PBS. Cells were subsequently lysed in 10mM TrisCl, pH 7.4, 10 mM NaCl, 3 mM MgCl_2_, 0.5% (V/V) NP-40. The run-on reactions were then carried out as described previously [29]. All signals were normalized to β-actin.

### 2.4. Small RNA Enrichment and End Label Small RNA Filter Blots

Small RNA was enriched from total RNA samples by filtration through YM-30 columns (Millipore). 1-2 milligrams of small RNA samples were end labeled with ^32^P g-ATP in a reaction using T4 Kinase (Invitrogen). Samples were hybridized to dot blot membranes as prepared for nuclear run-on in Ultra Hyb oligo buffer (Ambion) at 50 °C overnight. Two 15 minute washes were carried out in 0.1% SDS, 2× SSC at 50 °C. All signals were normalized to miR-16 [30].

### 2.5. Knockdowns

Lentiviral transduction particles were purchased from Sigma. MDA-MB-231 cells were infected according to manufacturer instructions. Cells were grown in 1.5 milligram/milliliter puromycin containing media after infection to maintain knockdown. Knockdown was verified by qRT-PCR as described above.

*Dicer* Catalog # NM_0306212-1322s1c1; *PIWIL4* catalog # NM_152431 1-1403s1c1; *Drosha* catalog #NM_0132352-4340s1c1

Sodium Bisulfite Conversion of DNA and Sequenom Analysis Genomic DNA was bisulfite converted using the EZ DNA Methylation Gold Kit (Zymo Research). The *BRCA1* promoter was amplified from converted DNA in a PCR reaction multiple products. 100 nanograms of PCR product was submitted for a sequenom analysis through the University of Arizona Genetics Core. Data was analyzed using the EpiTyper software (Sequenom).

### 2.6. Plasmid Generation and Nucleofections

SINE plasmid inserts were generated using PCR primers with appended restriction enzyme recognition site tails and cloned in p-Tracer SV-40 (Invitrogen) using standard cloning techniques. Nucleofection was accomplished according to Amaxa guidelines for cell line WI-38. Statistics were done using student’s t-test for unpaired samples.

### 2.7. Determination of Hybridization Stringency Conditions

Oligos were engineered with various degrees of identity (ranging from 65%–100%) to *laminB2*, a unique sequence in the human genome, and made into double stranded *in vitro* transcription templates using a Klenow extension reaction. Riboprobes were made using *in vitro* transcription and spotted onto nuclear run-on membranes. The transcription signal from nuclear run-on RNA was quantified for each of these probes.

## 3. Results and Discussion

### 3.1. Reduction in Steady State BRCA1 Transcript Levels is not Associated with Promoter Hypermethylation

**Figure 2:**
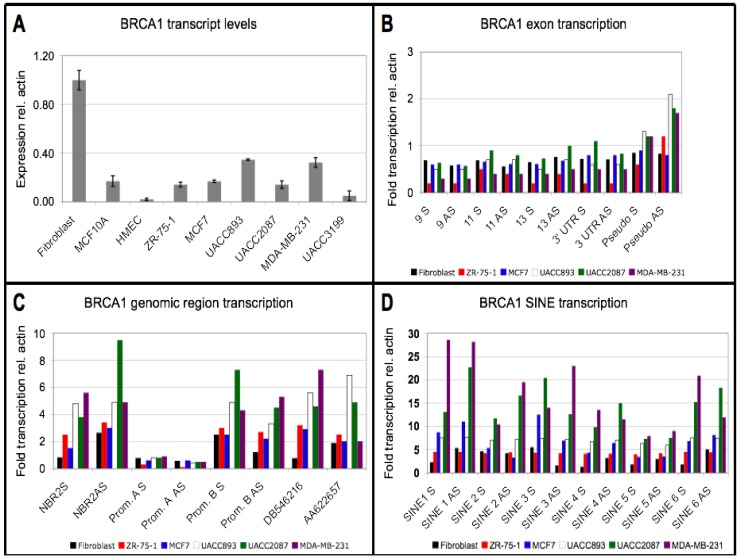
*BRCA1* and genomic region expression in cell lines. (A) *BRCA1* mRNA levels analyzed by qRT-PCR. Average and standard error of two biological replicates for each cell line are shown. (B) *BRCA1* transcription in fibroblast cell line and sporadic breast cancer cell lines. Probes 9, 11, 13, and 3’ UTR are from *BRCA1* exons and assay transcription solely from the *BRCA1* gene; pseudo probe assays transcription from exon one of both the *BRCA1* gene and the pseudogene. S detects transcription in the sense orientation, and AS detects transcription in the antisense orientation, relative to *BRCA1* transcription. (C) Transcription of other sequences within the *BRCA1* genomic region. NBR2: *NBR2* exon; Prom. A, bidirectional promoter shared between *BRCA1* and *NBR2*; Prom. B, alternative *BRCA1* promoter within first exon; DB546216 and AA622657, two ESTs transcribed from same sequence but in opposite orientations. The NBR2, Prom. B, DB546216 and AA622657 probes all contain SINEs. (D) Transcription of SINEs within *BRCA1* introns. Each probe spans 3–4 individual SINEs. Sense (S) and antisense (AS) refer to the SINE transcription orientation relative to the *BRCA1* coding transcript. See panel A for location of all probes in (B) through (D). Nuclear run-on data depicted in (B) through (D) represents one experiment with each cell line, although similar results were observed in other experiments using a subset of probes depicted here.

To establish a cell culture system to investigate potential mechanisms of *BRCA1* regulation, we searched for a set of established human cell lines exhibiting differences in *BRCA1* steady state transcript levels. We desired a control cell line that exhibited high levels of steady state *BRCA1* transcript compared to experimental cell lines. We additionally required control and experimental cell lines to exhibit comparable low levels of *BRCA1* promoter methylation, thus allowing us to investigate previously uncharacterized mechanisms of *BRCA1* regulation. *BRCA1* transcript levels were measured by quantitative reverse transcription PCR (qRT-PCR) in six breast cancer cell lines (MDA-MB-231, MCF7, UACC2087, UACC893, and UACC3199), two non-cancerous mammary epithelial cell lines (HMEC and MCF10A) and one fetal lung fibroblast cell line (WI-38 VA-13). Compared to all other cell lines, *BRCA1* mRNA was two to ten-fold higher in the fibroblast cell line (Figure 2A), making the fibroblast cells the most suitable control for our studies. The two regions of *BRCA1* analyzed (Figure 1A) showed similar expression profiles: region 1 spans exon 22 to the 3’ UTR, and region 2 spans exons 10 and 11. DNA methylation levels at the *BRCA1* promoter [31] were analyzed in all cell lines. UACC3199, which had previously been shown to exhibit methylation at the *BRCA1* promoter served as a positive control [32]. All the sporadic breast cancer cell lines showed very little methylation within the *BRCA1* promoter and did not differ significantly from the fibroblast cells (Figure S1). These data suggest that the observed low *BRCA1* transcript levels in the sporadic breast cancer cells are not associated with promoter hypermethylation.

While it would have been ideal to have a tissue specific control cell line, HMEC and MCF10A were not appropriate controls because of their low steady state *BRCA1* transcript levels. Previous reports indicated that *BRCA1* expression was higher in the HMEC line relative to sporadic breast cancer cells and that the promoter was relatively unmethylated [32], but this was not replicated, presumably because the *BRCA1* promoter became methylated during passage of the HMEC cell line. In addition, the extensive *BRCA1* promoter DNA methylation in HMEC (Figure S1) eliminated HMEC as a control, given that promoter methylation was likely to be regulating transcription of *BRCA1*. We found that, although *BRCA1* mRNA levels in MCF10A are comparable to the cancer cell lines (Figure 2), it is transcribed at a reduced rate in MCF10A cells compared to the fibroblast and cancer cell lines (Figure S2). These results imply that the *BRCA1* transcript is more stable in the MCF10A cells than in the breast cancer lines, raising the possibility that regulation of the *BRCA1* transcript occurs by different mechanisms in the MCF10A cells than in the breast cancer cell lines. Thus, MCF10A was also eliminated as a potential control cell line, as clear differences exist in regulation of *BRCA1* between these cells and the cancer cell lines.

### 3.2. Downregulation of BRCA1 Transcript Levels is not Associated with a Reduced Transcription Rate

We wished to determine whether the differences in steady state *BRCA1* transcript levels between the fibroblast cells and the sporadic breast cancer cell lines was due to differences in transcription. We performed nuclear run-on analysis to investigate transcription of *BRCA1* specific sequences in the fibroblast cell line and sporadic breast cancer cell lines. We observed no obvious difference in the transcription rate of *BRCA1* between the breast cancer cell lines and the fibroblast control cell line (Figure 2B), despite 2 to 10-fold differences (*P* < 0.01, Student’s t-test assuming unequal variance) in *BRCA1* steady state mRNA levels. These data suggest that differences in transcription are not contributing to the lower levels of *BRCA1* mRNA levels in the cancer cell lines relative to the fibroblast cell line. Thus, we hypothesized that *BRCA1* is likely to be downregulated at the post-transcriptional level in the sporadic breast cancer cells. Because the fibroblast cell line exhibited higher levels of *BRCA1* steady state mRNA compared to the sporadic breast cancer cell lines, yet had similar transcription rates and similar levels of promoter methylation, it was used as a control in all subsequent experiments to investigate post-transcriptional regulatory mechanisms resulting in the low levels of *BRCA1* mRNA in the cancer cell lines.

### 3.3. Repeat Sequences Are Highly Transcribed in Sporadic Breast Cancer Cell Lines

Although nuclear run-on experiments demonstrated that the rate of transcription of *BRCA1* exons was no different in control and cancer cell lines (Figure 2B), it remained possible that transcription of repeat sequences with sequence similarity to the introns or 3’ UTR of *BRCA1* could be upregulated. To investigate this idea, nuclear run-on transcription was analyzed using repetitive regions within and surrounding *BRCA1* as probes, including the introns that contain multiple SINEs. We observed that transcription from SINE sequences with high sequence similarity to those found within the *BRCA1* locus was increased in the sporadic breast cancer cell lines in both the sense (S) and antisense (AS) orientations compared to the fibroblast cell line (Figure 2C,D). We did observe a range of SINE transcriptional upregulation, with some breast cancer cell lines exhibiting comparable levels to the fibroblast and others showing dramatically higher levels. We do not find this surprising, however, as each cancer cell line was obtained from a different diseased individual and is therefore biologically distinct. However, we do observe the general trend that SINE transcription is increased in the breast cancer cell lines compared to the fibroblast cells.

A pair of intrachromosomal segmental duplications also occur within the *BRCA1* region: a 14-kb and a 24-kb duplication. After the original segmental duplication, other insertions occurred within the 24-kb duplication, thus explaining its larger size. The location of these duplications and how other nuclear run-on probes fall within them is depicted in Figure 1B and Figure S3A. We observed that transcription across the 14-kb intrachromosomal segmental duplication was elevated 2 to 30-fold (Figure S3B; location of probes relative to *BRCA1* shown in Figure 1B and Figure S3A); and transcription of SINEs with sequence similarity to those within *BRCA1* introns was upregulated 2 to 15-fold (Figure 2D). Other probes containing SINE sequences (Promoter B, NBR2, and DB546216/AA622657) were upregulated 2 to 8 fold (Figure 2C). We conclude that multiple repetitive regions, including SINEs, are transcribed at high levels in sporadic breast cancer cells relative to the control fibroblast cells. A similar trend was observed in other nuclear run-on experiments: high transcriptional signal was observed from SINEs in the breast cancer cell lines, while comparatively low transcriptional signal was observed from the same probes in control cells. The data we present is from one representative experiment. Interestingly, we observe similar levels of transcription in both the sense and antisense orientation from all probes analyzed, including *BRCA1*. However, this is not surprising, given that transcript profiling has revealed the presence of high amounts of both sense and antisense transcripts from protein coding genes in many organisms, including humans [33].

Because many of the repeats within *BRCA1* introns are SINEs and these SINEs have significant sequence similarity to large numbers of SINEs throughout the genome, we tested the stringency of our hybridization conditions and determined that they allowed for detection of transcripts with approximately 85% sequence identity or higher to our probes (Figure S4). A bioinformatic query of the entire human genome indicated that each SINE probe we used, which had 100% sequence identity to the *BRCA1* SINEs, could also detect transcripts from a substantial number of additional sequences throughout the genome. Non-*BRCA1* associated SINE sequences ranged from approximately 150 to 180,000 different SINEs, assuming a minimum length of 250 bp and a minimum of 85% sequence identity (Table S1). Thus, it was not possible to identify the specific location(s) from which SINE transcription was occurring. Given the substantial transcription levels observed, it is likely multiple locations are being transcribed.

### 3.4. Small RNA Species from BRCA1 and Associated Retrotransposons Are Elevated in Breast Cancer Cell Lines

**Figure 3:**
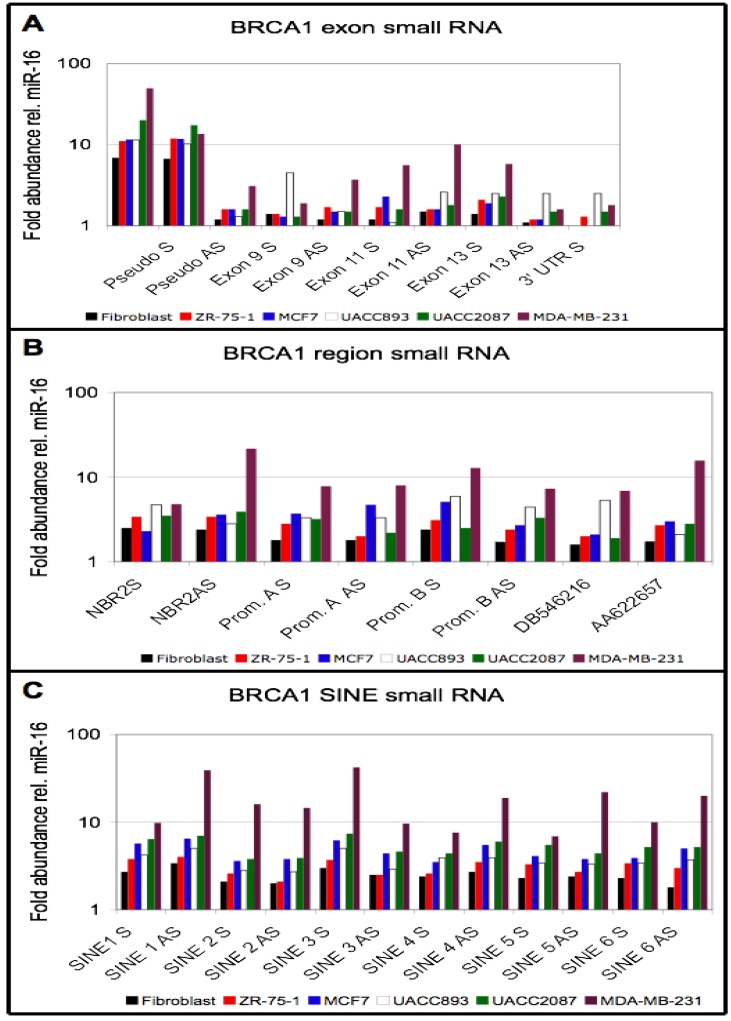
*BRCA1* region and SINE small RNAs in control and cancer cell lines. Probe nomenclature described in legend of Figure 1 and location of probes are indicated in Figure 1A. (A) *BRCA1* pseudogene and exons; (B) sequences surrounding and within *BRCA1*; and (C) SINEs within *BRCA1* introns. Value for 3’ UTR AS from fibroblast is negative and 1 for MCF7. Data presented represents one experiment with each cell line.

The observation that both sense and antisense transcription was detected from SINEs with high sequence similarity to those found within the *BRCA1* locus (Figure 2D) suggested that double-stranded RNA to SINE sequences might be present and potentially processed into small RNAs. To investigate this hypothesis, the abundance of small RNAs complementary to probes derived from SINE sequences within the *BRCA1* genomic region were compared between the breast cancer cell lines and the fibroblast cell line. We enriched for RNAs 60 nucleotides or smaller, end-labeled this small RNA enriched population with ^32^P g-ATP and used it to probe membranes containing the same target sequences used for the nuclear run-on analyses (see Figure 1A for probe locations). Small RNA species from both the sense (S) and antisense (AS) strand from multiple regions within the *BRCA1* genomic locus (Figure 3A,C), SINEs with high sequence identity to those in the *BRCA1* introns, and repetitive sequences within the 14 kb segmental duplication (Figure S3C), were elevated in the sporadic breast cancer cell lines. The observation that *BRCA1* exon-specific (non-repeat) small RNAs were also increased 2 to 6-fold in several of the breast cancer cell lines relative to the fibroblast cell line (Figure 3A) was consistent with a post-transcriptional RNA degradation mechanism contributing to the lower mRNA levels observed in the breast cancer cell lines.

To eliminate the possibility that the small RNA blots simply assayed degradation fragments of intact transcripts, levels of small RNA from the *GAPDH* and *ubiquitin* messages, which contain no SINEs within their sequences, were analyzed within the same samples used to measure *BRCA1* and SINE small RNAs and compared between cell lines. With the exception of MDA-MB-231, small RNA levels of these transcripts did not vary greatly between the cell lines (Figure S5). In all cases, the small RNAs from both *BRCA1* exonic sequences and the SINE sequences were higher in the cancer cell lines relative to the fibroblast than the small RNAs from *GAPDH* and *ubiquitin* genes.

### 3.5. BRCA1 Downregulation Occurs Through Dicer and Drosha

**Figure 4:**
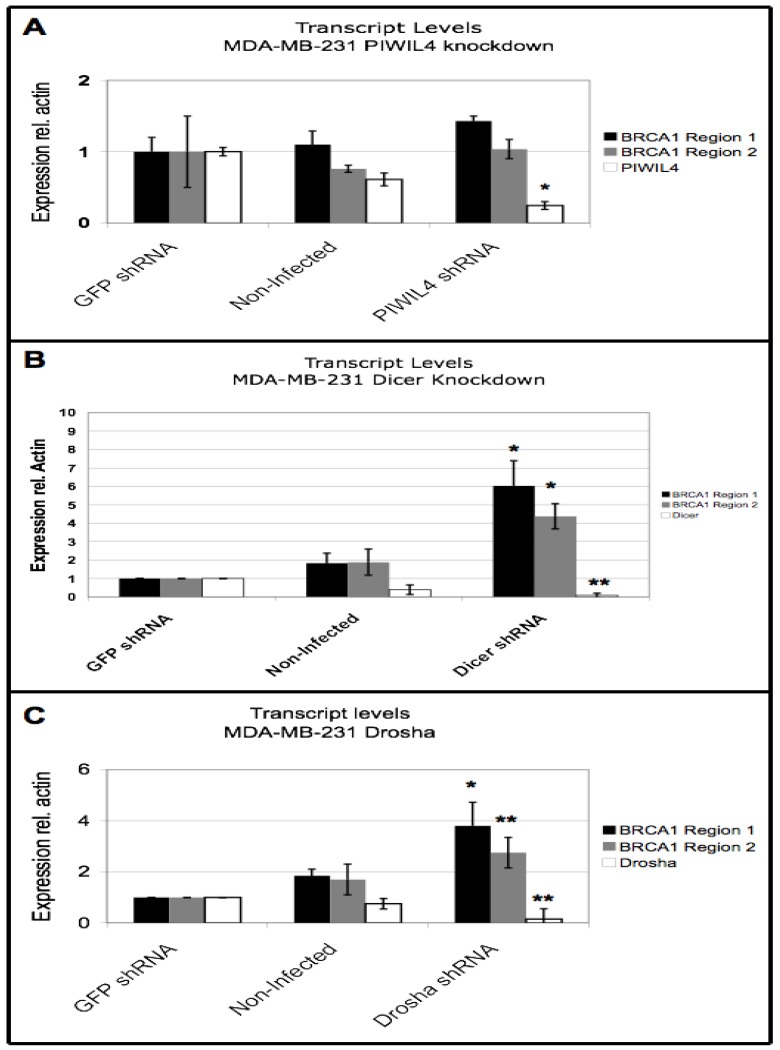
*BRCA1* downregulation requires *Dicer* and *Drosha* but not *PIWIL4*. (A) *PIWIL4* and *BRCA1* expression in *PIWIL4* knockdown MDA-MB-231 cells, non-infected cells, and GFP shRNA infected cells. Data shown is average and standard error of *BRCA1* and *PIWIL4* expression in two independent infections with *PIWIL4* shRNA lentivirus. P-value comparing *PIWIL4* expression in GFP shRNA infected cells and *PIWIL4* knockdown cells is 0.04. (B) *Dicer* and *BRCA1* expression in *Dicer* knockdown MDA-MB-231 cells, non-infected cells, and GFP shRNA infected controls. Data shown is average and standard error of *BRCA1* and *Dicer* expression in four independent infections with *Dicer* shRNA lentivirus. P-values comparing expression levels of *BRCA1* between GFP shRNA treated cells and *Dicer* shRNA treated cells: value for region 1 is 0.03, value for region 2 is 0.015. P-value comparing *Dicer* expression in GFP shRNA infected cells and *Dicer* knockdown cells is 0.002. (C) *Drosha* and *BRCA1* expression in *Drosha* knockdown MDA-MB-231 cells, non-infected cells, and GFP shRNA infected controls. Data shown is average and standard error of *BRCA1* and *Drosha* expression in six independent infections with *Drosha* shRNA lentivirus. P-values comparing expression levels of *BRCA1* between GFP shRNA treated cells and *Dicer* shRNA treated cells: value for region 1 is 0.014, value for region 2 is 0.005. P-value comparing *Drosha* expression in GFP shRNA infected cells and *Drosha* knockdown cells is 2.5 × 10^−6^. In each experiment, expression of the target gene within each cell line was normalized to actin and the GFP shRNA cells were set to 1.

The correlation of low *BRCA1* mRNA levels with increased small RNA species from both *BRCA1*-specific sequences and the repetitive SINE sequences located within *BRCA1* and elsewhere in the genome, suggested RNA processing pathways may be involved in downregulation of *BRCA1*. To test whether known post-transcriptional RNA regulation pathways might play a role in downregulating *BRCA1* transcript levels, the cancer cell line MDA-MB-231 was treated with lentiviral particles containing short hairpin RNA (shRNA) constructs targeting specific RNA processing enzymes. The cells were individually treated with lentiviral constructs targeting *PIWIL4*, *Drosha*, and *Dicer* to test for possible roles of the PIWI interacting RNA (piRNA), microRNA (miRNA), and small inhibitory RNA (siRNA) pathways in *BRCA1* regulation. Controls included non-infected cells and cells infected with a lentiviral shRNA construct against GFP, which does not target any sequence in the human genome. The same two regions of *BRCA1* were analyzed as described above (Figure 2A). Successful knockdown of all three RNA processing genes was achieved (Figure 4). Because expression levels of *PIWIL4*, *Dicer*, and *Drosha* (Figure 4) and four other RNA processing enzymes (Figure S6) were affected in GFP shRNA infected cells compared to non-infected cells, expression levels of the enzymes targeted by shRNA and those of *BRCA1* in the knockdown cells were normalized to GFP shRNA infected control cells.

While there was no significant effect on *BRCA1* transcript levels in *PIWIL4* knockdown cells (Figure 4A), in *Dicer* knockdown cells *BRCA1* transcript levels were significantly increased by an average of 6-fold (standard error 1.5, *P* < 0.04, Student’s t-test assuming unequal variance) across four biological replicates (Figure 4B). Similarly, in *Drosha* knockdown cells *BRCA1* mRNA levels were increased by an average of 4-fold (standard error 1, *P* < 0.02, Student’s t-test assuming unequal variance) across six biological replicates (Figure 4C). The two analyzed regions of *BRCA1* consistently behaved differently in degree of rescue; therefore, both are shown individually (Figure 4). Together, these results suggest that both *Dicer* and *Drosha* contribute to downregulation of *BRCA1* transcripts in the sporadic breast cancer cell lines.

### 3.6. Ectopic Expression of SINE Sequences Induces Downregulation of BRCA1 in Non-Cancer Cells

**Figure 5:**
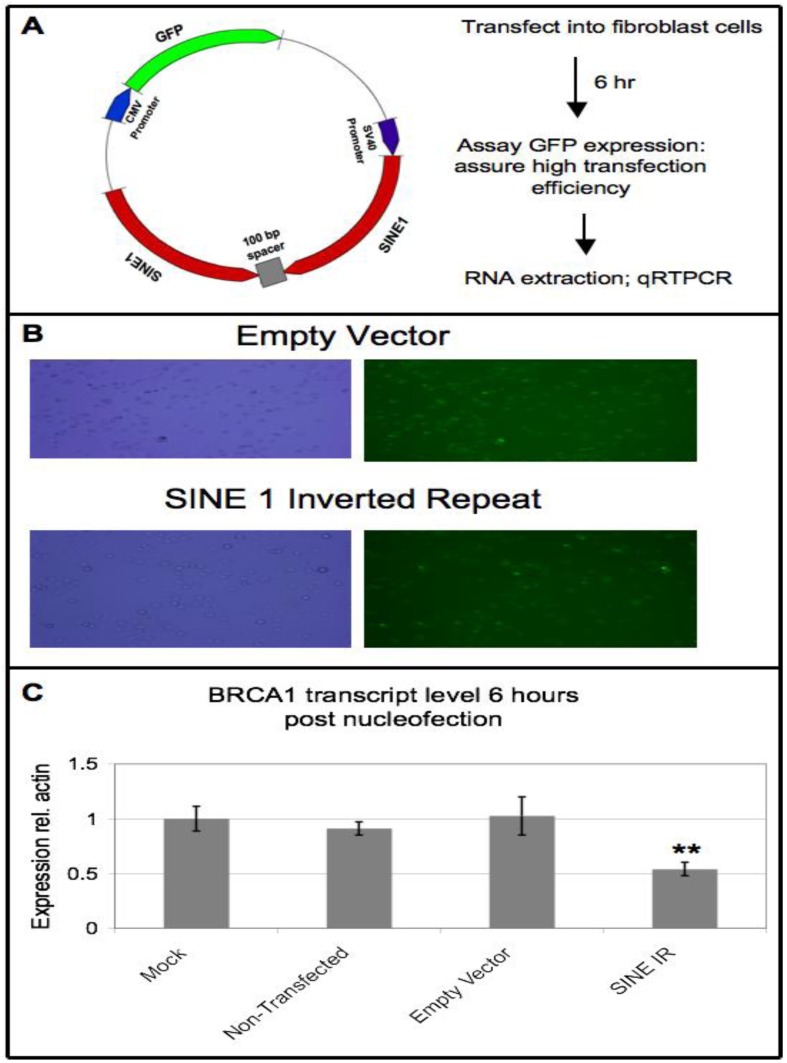
Ectopic expression of SINE sequences induces downregulation of *BRCA1*. (A) Diagram of plasmid and experimental strategy. An inverted repeat to the SINE 1 probe used in the run-on and small RNA blots, which is 100% identical to the SINE located within *BRCA1* intron 3, was cloned under the control of the highly expressing SV40 promoter. Within the same plasmid, GFP is under the control of the highly expressing CMV promoter. (B) Transfection efficiency as shown by number of cells in bright field (left) exhibiting GFP expression (right). Upper image shown is cells treated with the empty vector 6 hours post transfection; lower image is one biological replicate of SINE 1 inverted repeat 6 hours post transfection. (C) *BRCA1* transcript levels 6 hours post-transfection, as determined by qRT-PCR. Data shown is average and standard error of four biological replicates. P-value for *BRCA1* expression between the mock treatment and treatment with the SINE 1 IR is 0.004.

To determine whether increased dsRNA from SINE sequences could downregulate *BRCA1* mRNA levels in non-cancer cells, transient transfection assays were used in the fibroblast cells to express an inverted repeat of the SINE 1 probe sequence, which is 100% identical to a SINE within *BRCA1* (Figure 5A). Transfection efficiency, as observed by GFP expression, was close to 100% (Figure 5B). Cells transfected with the SINE inverted repeat construct showed 2-fold, (standard error 0.1, *P* = 0.004, Student’s t-test assuming unequal variance) lower *BRCA1* mRNA levels, measured by qRT-PCR, as compared to the mock treatment (Figure 5C). We repeat this experiment in two other human cell types, HeLa and HEK293-T cells; however, we observed no reduction of *BRCA1* mRNA levels upon SINE inverted repeat transfection (data not shown). These results may indicate that SINE mediated knockdown of *BRCA1* is a tissue specific phenomenon of gene regulation. Transfection of fibroblast cells with plasmids expressing sense or antisense SINE sequence, relative to the *BRCA1* transcript, resulted in no significant alterations in *BRCA1* mRNA levels (Figure S7). This is not surprising as a review of the literature indicates that hairpin RNA is a much more efficient silencer than sense or anti-sense RNA [34,35]. Of the ~150 additional human genes identified to contain SINEs with high sequence identity to the SINE 1 probe within their introns, four were tested for downregulation upon transfection with the SINE 1 inverted repeat, but none of these genes were downregulated six hours post transfection (Figure S8). This result could be because the similarity was not 100% to the SINE 1 sequence (Figures S9-S12), reducing the number of small RNAs with perfect complementarity to each target gene. In contrast, each small RNA generated from the SINE 1 inverted repeat would perfectly match the *BRCA1* transcript. Thus, it may be that a certain threshold in the number of SINE small RNAs that retain a perfect or near perfect match to a target gene may be required to effectively trigger transcript degradation.

Examination of transcription rates of *BRCA1* revealed that although breast cancer lines had lower steady state levels of *BRCA1* mRNA, they did not differ in *BRCA1* transcription rate when compared to each other and to fetal lung fibroblast, which had a much higher level of *BRCA1* transcripts. This suggests a post-transcriptional mechanism is operating to downregulate *BRCA1* in the breast cancer cell lines. We cannot exclude the possibility that this post-transcriptional mechanism is due to differences in the tissue type of our control fibroblast cells when compared to breast cells rather than the transformed nature of the cancer cell lines. However, SINE mediated transcript regulation in human cells is an exciting and novel observation, regardless of whether it occurs in healthy *vs.* diseased states or in a tissue specific context. Our observations nevertheless suggest a novel RNA-mediated post-transcriptional silencing mechanism in which SINE upregulation (likely throughout the genome) is contributing to post-transcriptional targeting of *BRCA1* RNA. Given the correlation with increased transcription of both the sense and antisense orientation from SINEs, a reduction in *BRCA1* steady state transcripts and increased small RNAs homologous to SINE sequences and *BRCA1*-specific sequences, we hypothesize the increased SINE transcripts and small RNAs are triggering *BRCA1* RNA degradation. Consistent with this hypothesis, the low *BRCA1* mRNA levels could be rescued by depleting *Dicer* and *Drosha* functions. Our experiments cannot distinguish whether *Dicer* and *Drosha* are acting directly on the *BRCA1* RNA or whether the miRNA and siRNA pathways or solely the miRNA pathway is required. However, the observation that SINE inverted repeat expression can induce *BRCA1* downregulation is most consistent with a siRNA mechanism, as the small RNAs generated from the inverted repeat would target intronic *BRCA1* sequences.

We propose a model whereby *BRCA1* can be regulated post-transcriptionally by a pathway initiated through increased bidirectional transcription of SINEs or production of sense and antisense SINE RNA from different loci. This increased SINE transcription, which is likely to occur at hundreds or thousands of sites throughout the genome, may generate dsRNA that subsequently could be processed into SINE small RNAs. *BRCA1* has a high density of SINEs within its introns, and SINE small RNAs could target the *BRCA1* transcript for degradation in *trans*. It is important to stress that the SINEs from which transcription is highest may or may not be those within the *BRCA1* locus: small RNAs originating from SINEs at distant genomic regions could target the SINE dense *BRCA1* transcript via sequence identity. Our results demonstrating that expression of a SINE hairpin RNA is sufficient to decrease in *BRCA1* transcript levels is consistent with a *trans*-acting model.

Our findings suggest a novel mechanism might be governing the regulation of *BRCA1* transcripts. The observation that non-cancer fibroblast cells can be induced to specifically downregulate *BRCA1* steady state mRNA levels by expressing a SINE inverted repeat suggests that SINE transcription and subsequent generation of dsRNA and small RNAs could target protein coding transcripts in human cells. Whether the postulated SINE RNA destruction of primary transcripts is limited only to disease states where endogenous SINE transcription is upregulated, as in the breast cancer cell lines, is currently unknown. In support of the hypothesis that retrotransposon derived small RNAs can target protein coding genes, recent reports show that piRNAs can target 3’UTRs of protein coding transcripts in *Drosophila* and *Xenopus* [36]. Additionally, transcripts that contain *Alu* elements within their 3’UTR can be targeted by long non-coding RNAs (lncRNAs) with complementary *Alu* sequences for the STAU-1 RNA decay pathway [37]; notably, this model is similar to what we propose in that one *Alu* containing lncRNA can target a variety of protein coding transcripts containing sequence homology provided by *Alu* elements within their 3’ UTR.

## 4. Conclusions

Given the tremendous number and sequence divergence of different SINE families within the human genome, we speculate that controlled transcription of specific SINE family members could have evolved as a gene regulation mechanism for human genes that, like *BRCA1*, contain large numbers of intronic and 3’-UTR SINE sequences. Further, in diseases where SINE deregulation has been known to occur, such as cancer, it may be an unexplored mechanism contributing to loss of gene expression attributed to the disease phenotype.

## Supplementary Files

Supplementary File 1 Supplementary Materials (PDF, 1256 KB)
