# Supplementary material for: High SINE RNA Expression Correlates with Post-Transcriptional Downregulation of BRCA1
Source: Genes (Basel). 2013 Apr 29;4(2):226–43. doi: 10.3390/genes4020226 (PMC3899967; doi:10.3390/genes4020226)

**Table S1.** Characteristics of SINE probes used in nuclear run-on and end-labeled small RNA blot experiments.

| Probe | Number of Hits Genome-Wide (>/=250 bp, >/=85% Sequence Identity) | Highest Percent Identity Genome-wide, non-BRCA1 |
|---|---|---|
| SINE1 | 143 | 89.6 |
| SINE2 | 3693 | 89.4 |
| SINE3 | 121,177 | 91.3 |
| SINE4 | 95,322 | 91.5 |
| SINE5 | 105,706 | 92.1 |
| SINE6 | 178,780 | 96.1 |

**Table S2.** Primers used in qRT-PCR experiments.

| Primer Name | Sequence |
|---|---|
| BRCA1 Region 1 F | GTGGTGCTTCTGTGGTGAAG |
| BRCA1 Region 1 R | ACAGGTGCCTCACACATCTG |
| BRCA1 Region 2 F | GGCTATCCTCTCAGAGTGACATT |
| BRCA1 Region 2 R | CTGATGTGCTTTGTTCTGGA |
| PIWIL4 F | TGCTGATGTGAGTTACAAAGTCC |
| PIWIL4 R | CCTATTAGCTGCTTCTCACACG |
| Drosha F | GGAATTAGGCACAGCATTTATCC |
| Drosha R | GAGACTGTGATCCGGTAGTGG |
| Dicer F | AAATACATGCACTATGTGAAGAGC |
| Dicer R | TCGCTCATATGGTTTATATTTGC |
| BAZ2B F | GTGGCTTCAGTAGTTTCAAAAGG |
| BAZ2B R | GACACTGTGGACAGGTTAAACG |
| DYNC1H1 F | GACTCTGGATATCTTGAAACATGG |
| DYNC1H1 R | AGAGGGAAATCTTTCATCAGAGG |
| SETD1A F | AAGATCGAGAGGAAGCTGTGG |
| SETD1A R | AGTCTGATGTGCTGTCATTTTCG |
| USP48 F | TAGTGACTACATGCTGGGAGACG |
| USP48 R | AATGTATCGCCTATTACTGTTTTGC |
| β-Actin F | TGGTGATGGAGGAGGTTTAGTAAGT |
| β-Actin R | AACCAATAAAACCTACTCCTCCCTTAA |

**Table S3.** Primers used in sequenom experiment.

| Primer Name | Sequence |
|---|---|
| BRCA1 Promoter A F | AGGAAGAGAGTAATTGGAAGAGTAGAGGTTAGAGGGT |
| BRCA1 Promoter A R | CAGTAATACGACTCACTATAGGGAGAAGGCTCCAAAACAAAAAATAAAAACCTCCT |

**Table S4.** Primers used to create templates for riboprobes synthesis for dot blot membranes.

| Primer Name | Sequence |
|---|---|
| Actin F | TCATGTTTGAGACCTTCAACACC |
| Actin R | CACAGCTTCTCCTTAATGTCACG |
| BRCA1 Exon 9 F | AATGTCACCTGAAAGAGAAATGG |
| BRCA1 Exon 9 R | TGCTAAAAACAGCAGAACTTTCC |
| BRCA1 Exon 11 F | ATAAAGCTCCAGCAGGAAATGG |
| BRCA1 Exon 11 R | TTTCTGATGTGCTTTGTTCTGG |
| BRCA1 Exon 13 F | ATAGGTGGTACATGCACAGTTGC |
| BRCA1 Exon 13 R | AGTAAGATGTTTCCGTCAAATCG |
| BRCA1 3’ UTR F | GAAGCTGTTGCTTTCTTTGAGG |
| BRCA1 3’ UTR R | GAAGACCTAGTCCTTCCAACAGC |
| Pseudo F | AGCTCGCTGAGACGTTCTGG |
| Pseudo R | CCCCTCGCATAAGAATACCC |
| NBR2 F | TTCACTGTGTTAGCCAGGATGG |
| NBR2 R | TGAGAAAACAGGCTTAAAAGAGG |
| AA622657/DB546216 F | TGAGACAGTTTGGCTCATTCC |
| AA622657/DB546216 R | CTTTGGGATCACTTGAGGTTAGG |
| BRCA1 Promoter A F | ACGGAAACCAAGGGGCTACC |
| BRCA1 Promoter A R | ATGCAATAAGCCGCAACTGG |
| BRCA1 Promoter B F | GAATCCTCGTGATAGGAACTGG |
| BRCA1 Promoter B R | GGTCCCATCCTCTCATACATACC |
| 14-1 F | CAGAAGAGGCATGATGTTTGG |
| 14-1 R | GCACCTTGACCTATCTGATGTCC |
| 14-2 F | TAATCCAGGAGGATATTGTAGGG |
| 14-2 R | CAATGTTGGTACAAGTTATCTCAGG |
| 14-3 F | AAGTCAAAAATGTTATAGTCATAGG |
| 14-3 R | TGTTTTCTAGCTTTTCATTGC |
| 14-4 F | GGTAATTTTAAATAGGGGTGTCTGG |
| 14-4 R | TTCATTGGAACAGAAAGAAATGG |
| 14-5 F | AAACCAAAGAACTAATGACAACG |
| 14-5 R | GACTGCTTTGGACAATAGGTAGC |
| 14-6 F | CAGCTAATCGTTTTAGTGACAGG |
| 14-6 R | CAAGAACAAAGCTTCCACAGC |
| 14-7 F | TAGCATTTATCTGCTGGTAACAC |
| 14-7 R | ATCGAGGTCATCCTGGCTAACG |
| 14-8 F | TATTTCAGAATATCCGCTTTTGG |
| 14-8 R | AAATGAGCTAAGCGCTACTTCG |
| BRCA1 SINE 1 F | GCAGACACTGACAATGAAGAGACC |
| BRCA1 SINE 1 R | ACCCAAGTCTCCTTGACCTTACC |
| BRCA1 SINE 2 F | TTTTTCTATTTCCAATCCCTTCTGC |
| BRCA1 SINE 2 R | CTGTTCCCTCCCCAGACAACC |
| BRCA1 SINE 3 F | TTTTTAAAAGATAGGGTCTCAGTCACC |
| BRCA1 SINE 3 R | TATAGGCCCTTCCTTGCTTTGG |
| BRCA1 SINE 4 F | CCAAATGCTTAAAAATATCAGTGACC |
| BRCA1 SINE 4 R | CCAAAGTATGGGCTACAGAAACC |
| BRCA1 SINE 5 F | GCTAACACTCAGTGATGAGGATGC |

| | |
|---|---|
| BRCA1 SINE 5 R | TAAGTGCTATGTTCCCAGTCTGC |
| BRCA1 SINE 6 F | AGGCAGGAGAATCTTTTGAATGC |
| BRCA1 SINE 6 R | TTACTTGCTTTTGGTCAAATCTCTGC |

**Figure S1.** BRCA1 promoter methylation in all cell lines. BRCA1 promoter methylation levels as analyzed by Sequenom. A total of 18 CpG residues across 317 bp spanning the promoter and first exon of BRCA1 and the first exon of NBR2 were analyzed; average and standard error for each cell line is shown. The analyzed sequence contains the 267 bp region shown to have optimal promoter activity in transient transfection experiments [28].

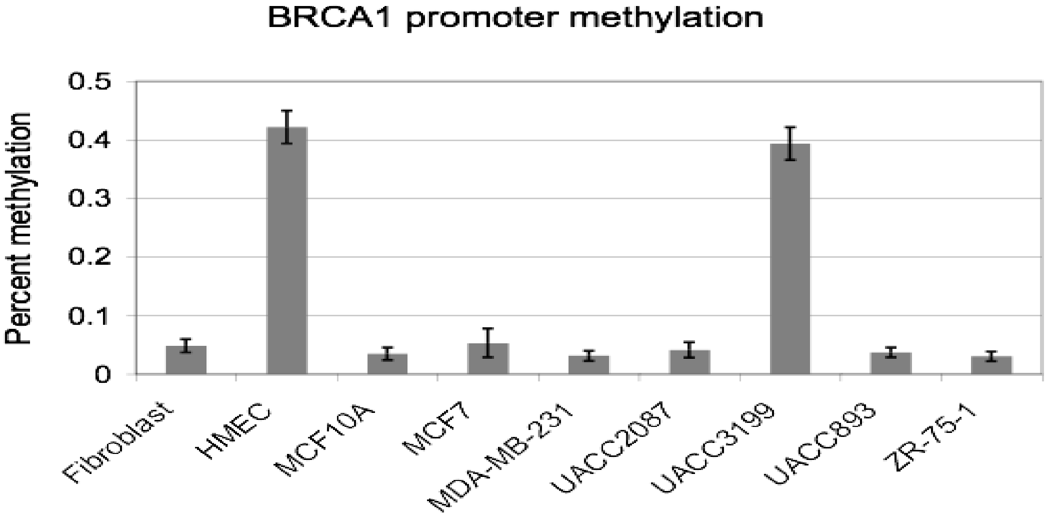


**Figure S2.** BRCA1 transcription rate is reduced in MCF10A cells. BRCA1 transcription in fibroblast cells, non-cancerous mammary epithelial cells MCF10A, and two sporadic breast cancer cell lines MCF7 and UACC893, as analyzed by nuclear run-on. Probes 9, 11, 13, and 3' UTR are from BRCA1 exons and the probes assay transcription solely from the BRCA1 gene; the pseudo probes assay transcription from exon one of both the BRCA1 gene and pseudogene. See Figure 1A for location of probes. S: sense; AS: antisense.

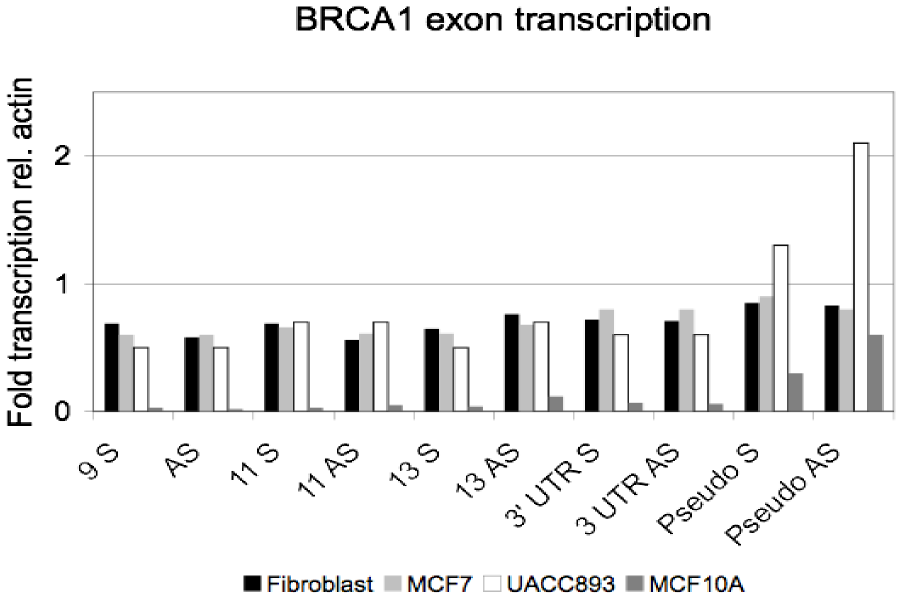

**Figure S3.** Transcription and small RNA from 14 kb segmental duplication. (A) Diagram of the BRCA1 genomic region and relative location of 14 kb and 24 kb intrachromosomal segmental duplications. Regions are not presented to scale. Location of 14 kb segmental duplication tiling probes and relative location of probes shown in Figure 1A that fall within this region are depicted. (B) Transcription of regions within the 14-kb segmental duplication as analyzed by nuclear run on in control and breast cancer cell lines. S: sense; AS: antisense. (C) Small RNA abundance from 14 kb segmental duplication in control and breast cancer cell lines. Small RNA levels are normalized to miR-16.

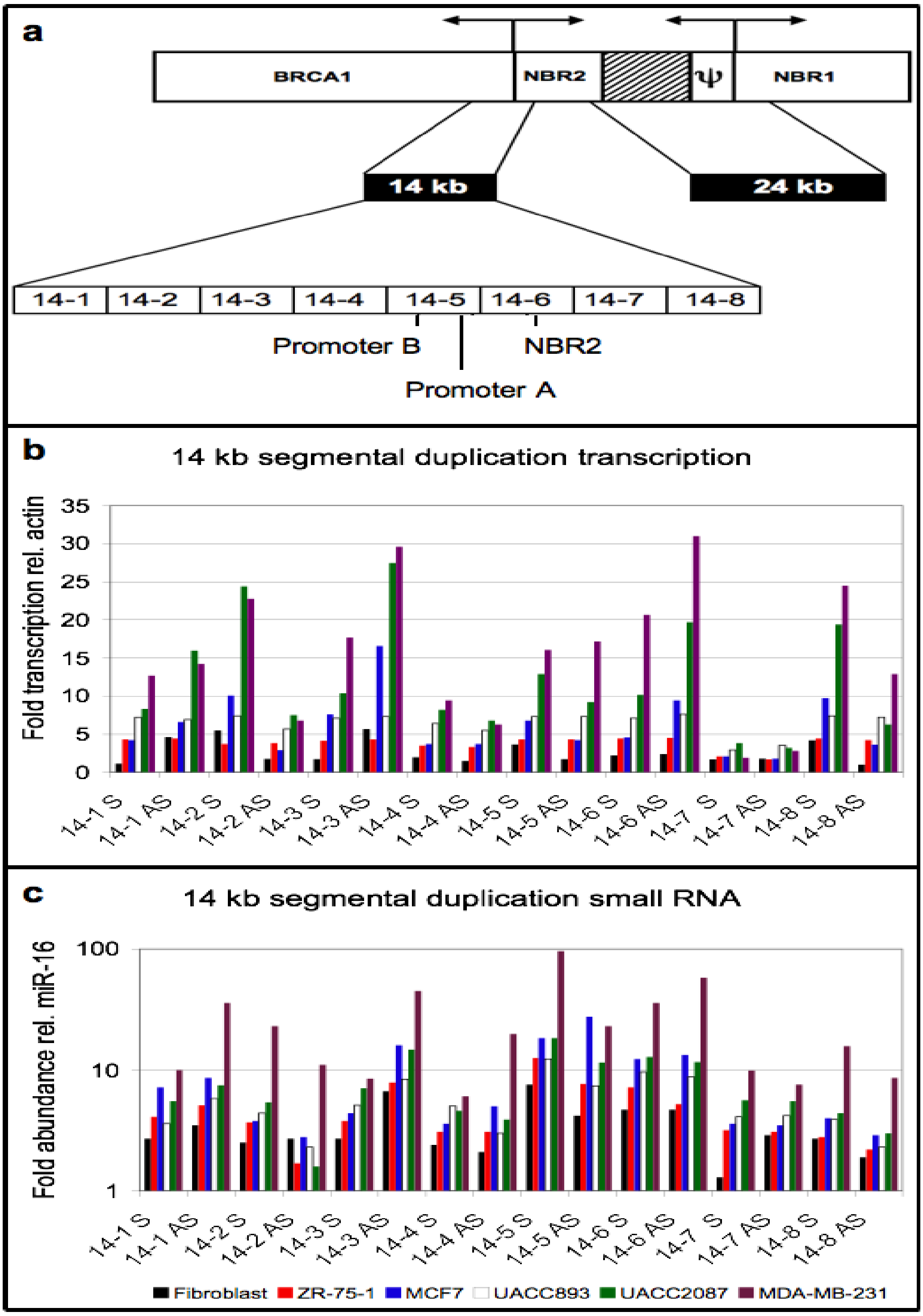

**Figure S4.** Stringency of hybridization conditions in nuclear run-ons. Oligos with a range of sequence identity to lamin B1 were engineered to contain sequence changes relative to the consensus sequence of lamin B1 and spotted onto nuclear run-on dot blot membranes as described. Six replicates of nuclear run-on experiments were performed, as described, and signal intensity of each probe was quantified by Quantity One Software (BioRad). Data presented is average and standard error of percent signal relative to the 100% identity probe for all six replicates. Percent signal of lambda DNA, present as a negative control, is presented to represent background signal levels.

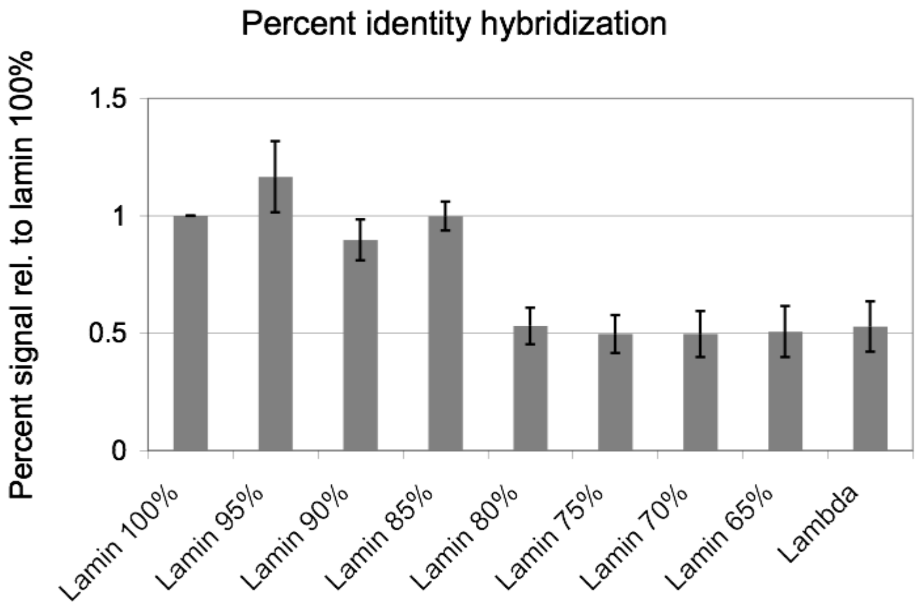


**Figure S5.** Small RNA levels of GAPDH and ubiquitin messages. Small RNA abundance of GAPDH and ubiquitin mRNAs were analyzed by small RNA fractionation followed by radioactive end-labeling and hybridization to dot blot membranes. Small RNA levels are normalized to microRNA 16 (miR-16).

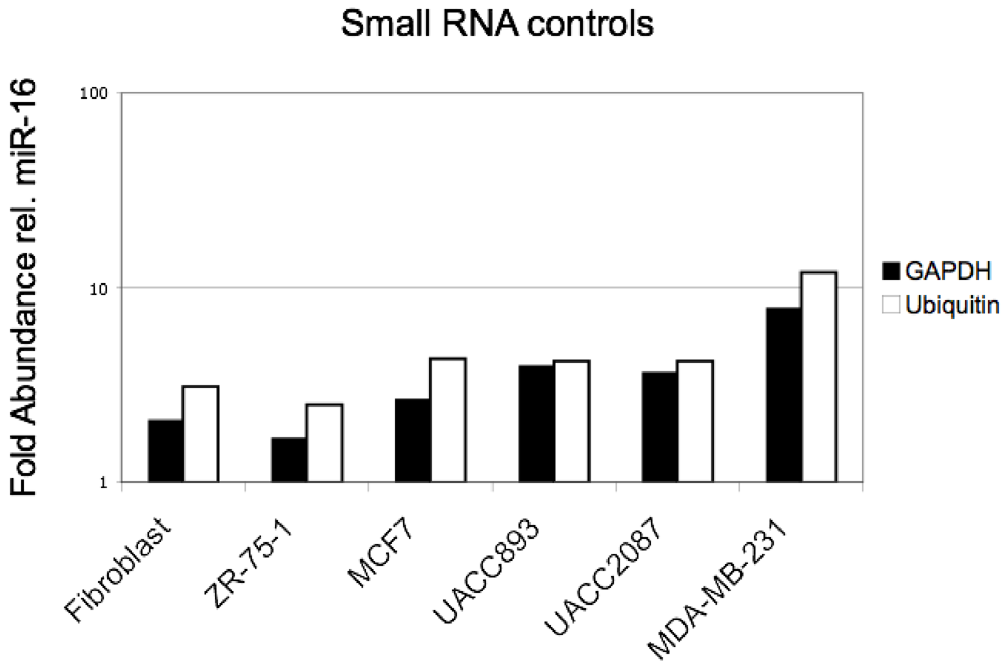

**Figure S6.** Expression of RNA processing enzymes in non-infected cells and GFP shRNA infected cells. Expression of indicated RNA processing enzymes in non-infected cells and GFP shRNA infected cells for (A) MDA-MB-231 cell line and (B) MCF7 cell line. Expression was analyzed by qRT-PCR and transcript levels for all enzymes were normalized to β-actin. Expression of all genes in the GFP shRNA infected cells was set to 1. Data shown is average and standard error for two technical replicates of each primer pair.

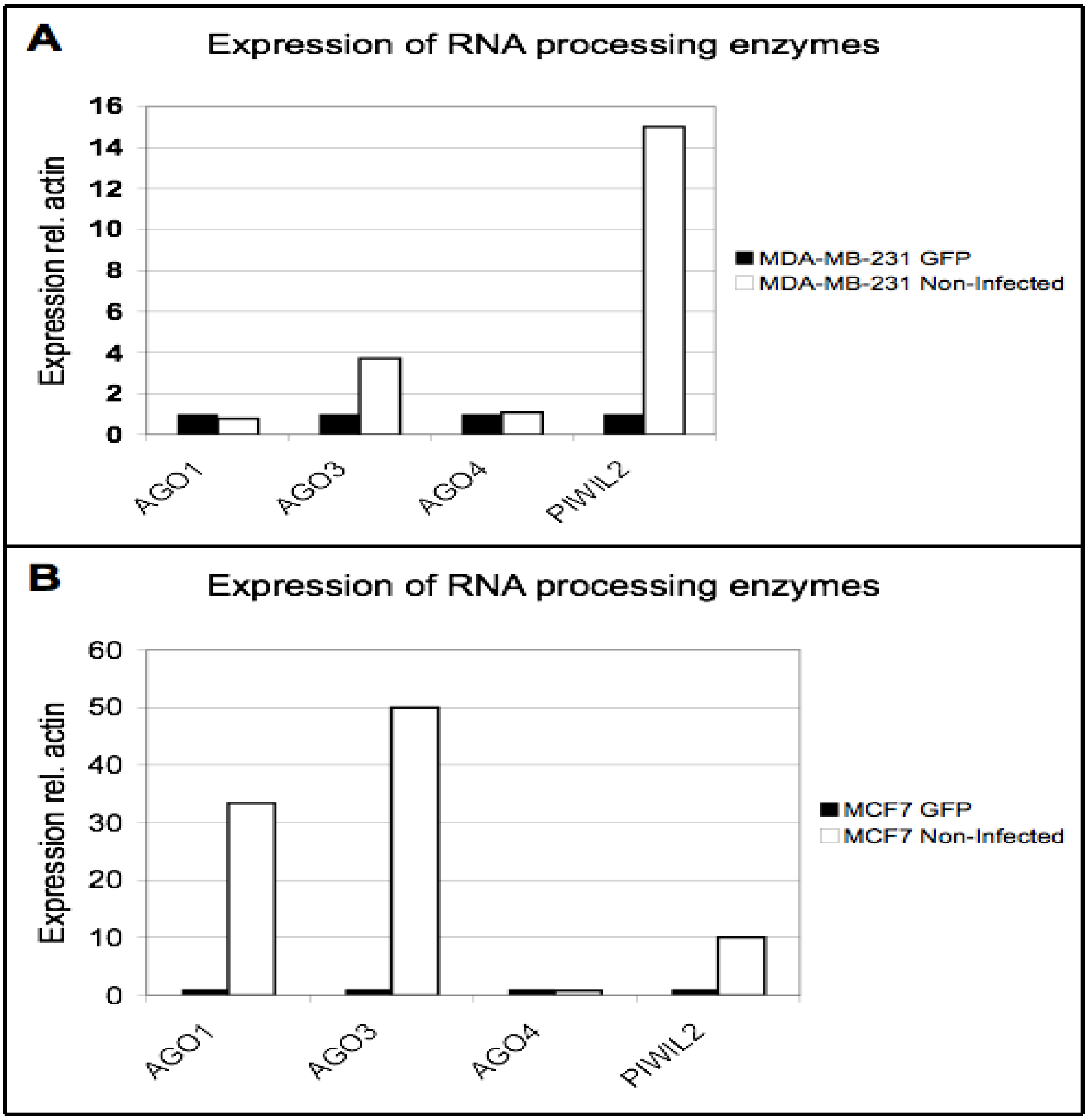

**Figure S7.** Effects of SINE sense/antisense expression on BRCA1 transcript levels. Expression of BRCA1 was determined 6 and 24 hours post-transfection of plasmids containing the SINE1 probe in either the sense or antisense orientation relative to the BRCA1 coding transcript. Non-transfected, mock and empty vector transfections serve as controls. Expression was normalized to β-actin and data is depicted relative to the mock treatment control, which was set to 1. Data depicted is average and standard error of two biological replicates.

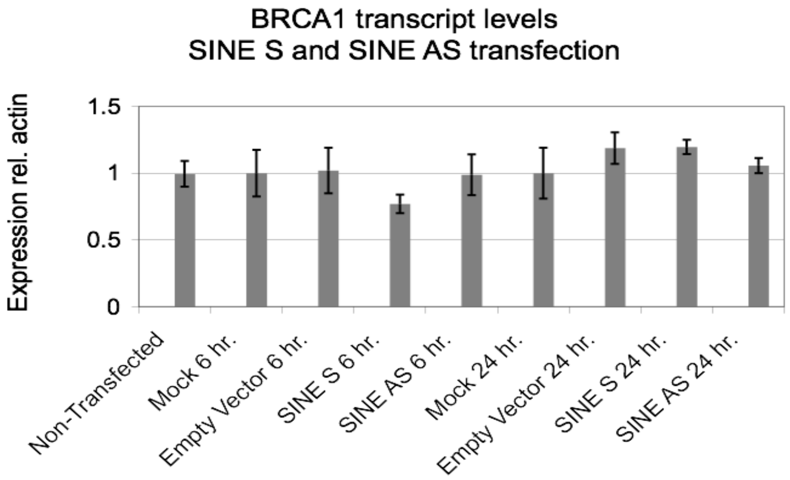


**Figure S8.** Expression of candidate genes for SINE dsRNA induced knockdown. Four genes containing SINE sequences homologous to the SINE 1 construct used in the transfection experiments within their introns were selected by a bioinformatics search and were defined as candidate genes for SINE 1 IR mediated knockdown. Expression level of all genes 6 hours post transfection was analyzed by qRT-PCR and normalized to β-actin. 3 independent replicates of inverted repeat transfection were compared to an empty vector transfection, a mock treatment, and non-transfected cells. Results for expression of BRCA1, as is shown in Figure 5, are depicted for the purpose of comparison. Data shown is average and standard error for two technical replicates of each primer pair.

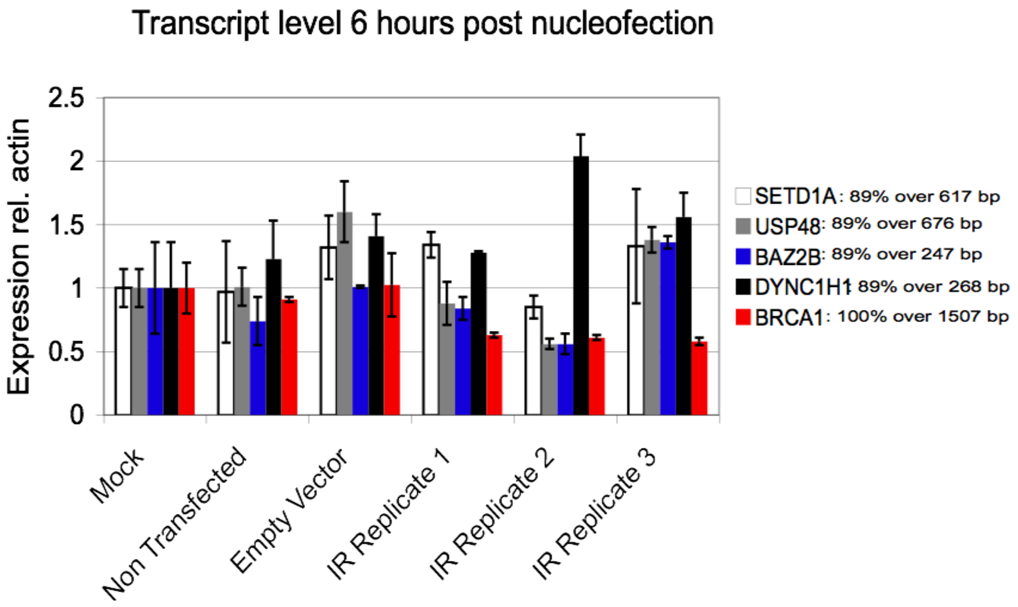

**Figure S9.** Alignment of BAZ2B to SINE1. SINE 1 sequence is cropped to show only relevant region BAZ2B. Identity is indicated by shading.

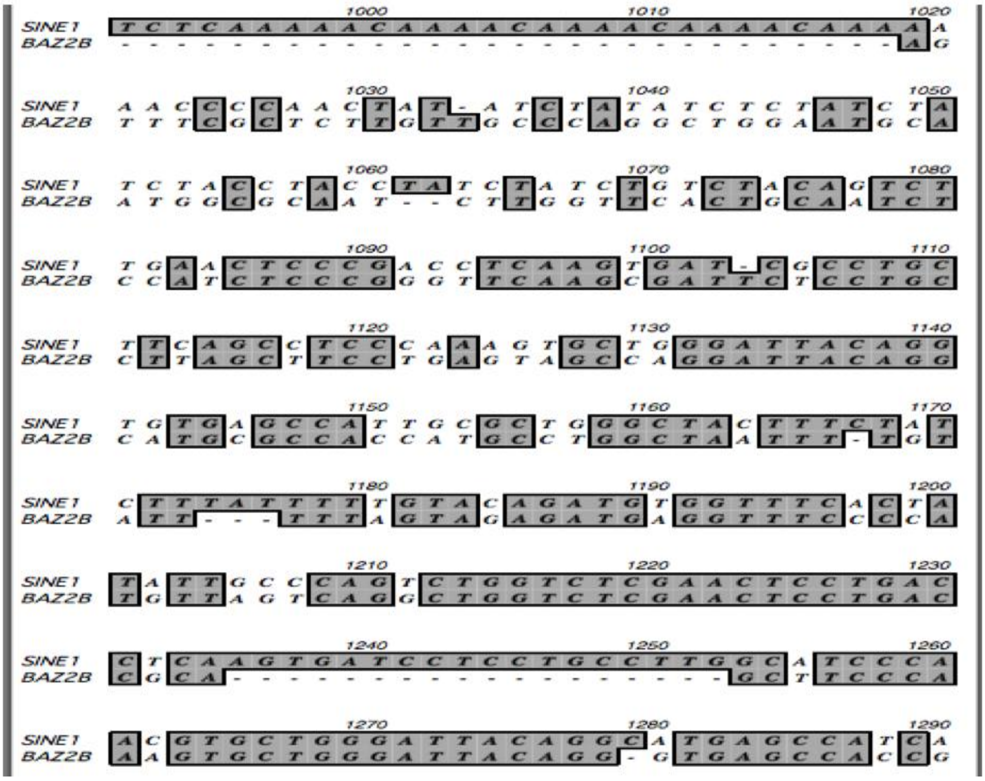

**Figure S10.** Alignment of DYNC1H1 to SINE1. SINE 1 sequence is cropped to show only relevant region DYNC1H1. Identity is indicated by shading.

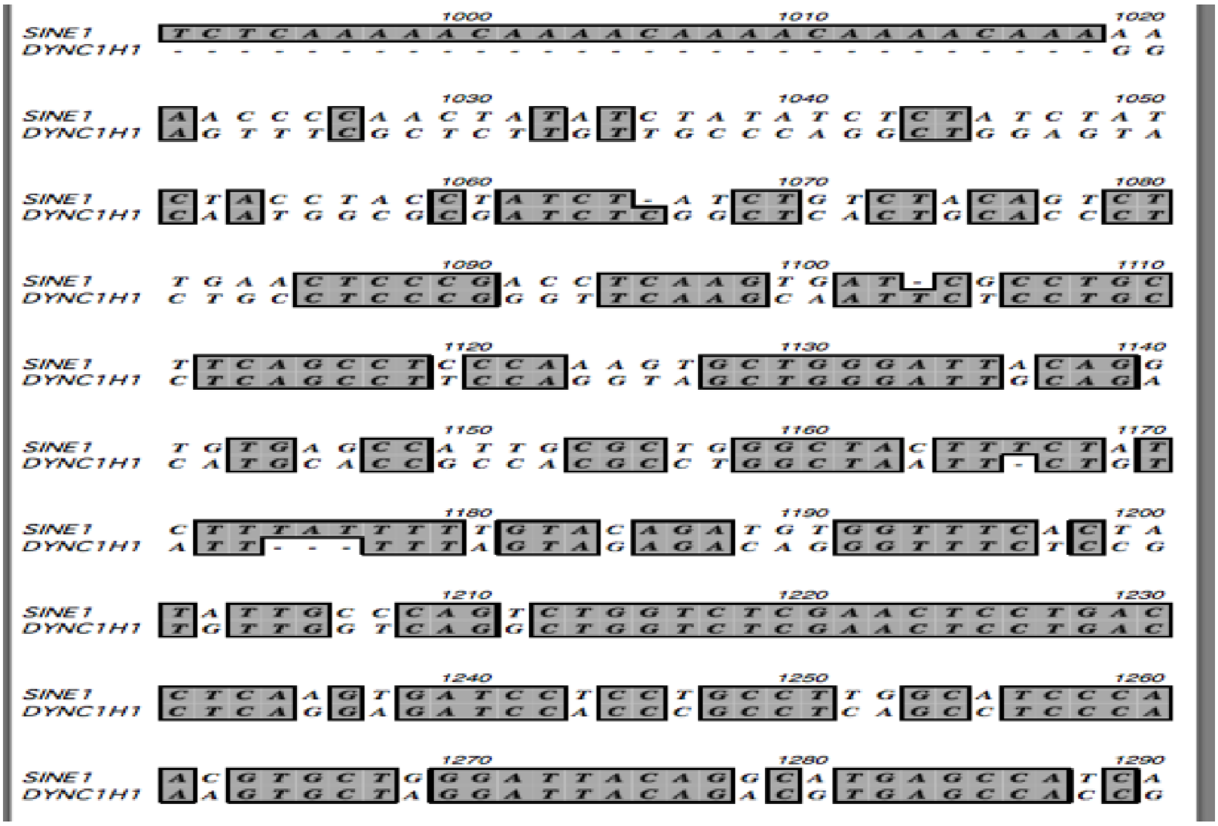

**Figure S11.** Alignment of SETD1A to SINE1. SINE 1 sequence is cropped to show only relevant region SETD1A. Identity is indicated by shading.

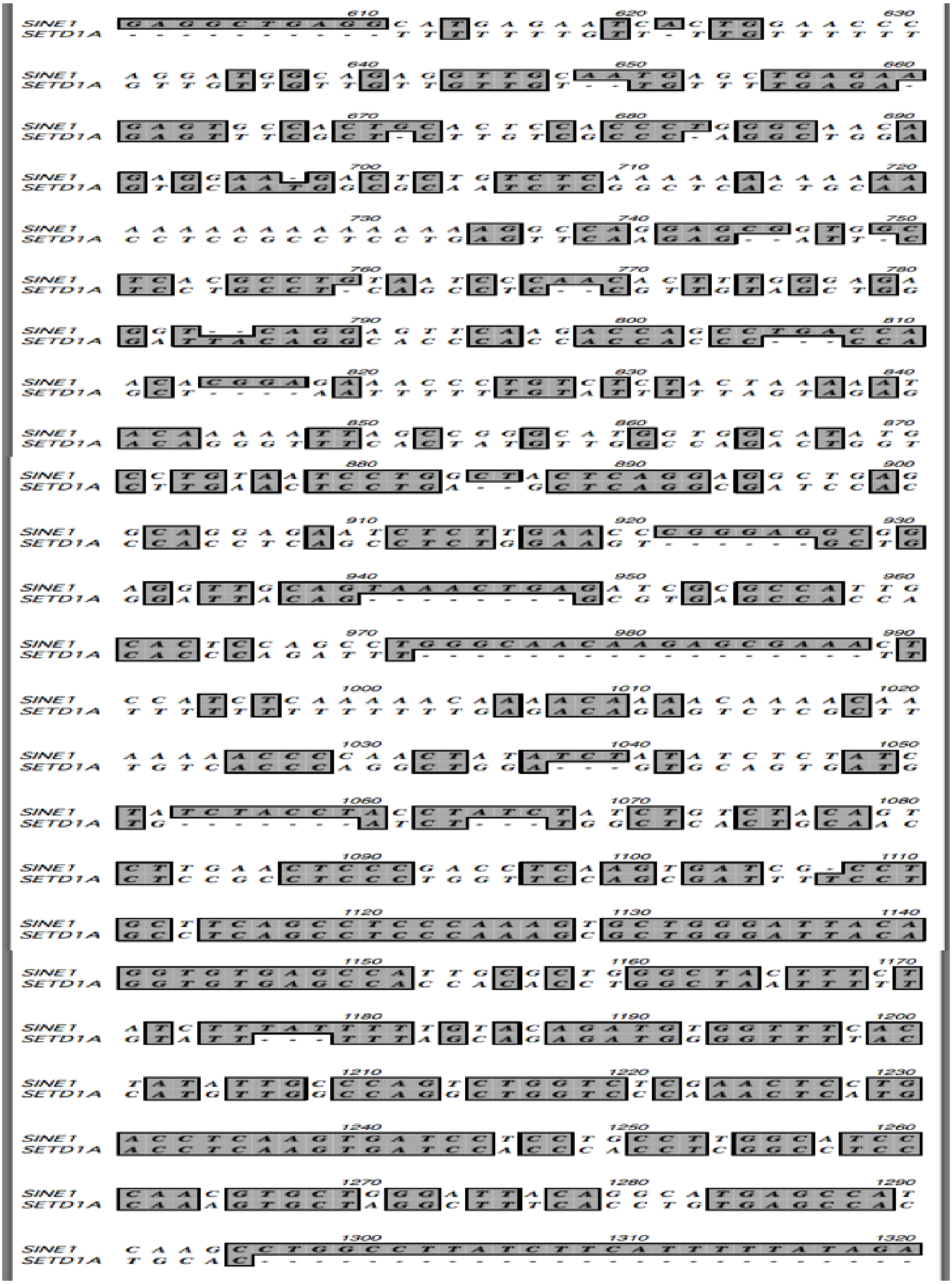

**Figure S12.** Alignment of USP48 to SINE1. SINE 1 sequence is cropped to show only relevant region USP48. Identity is indicated by shading.

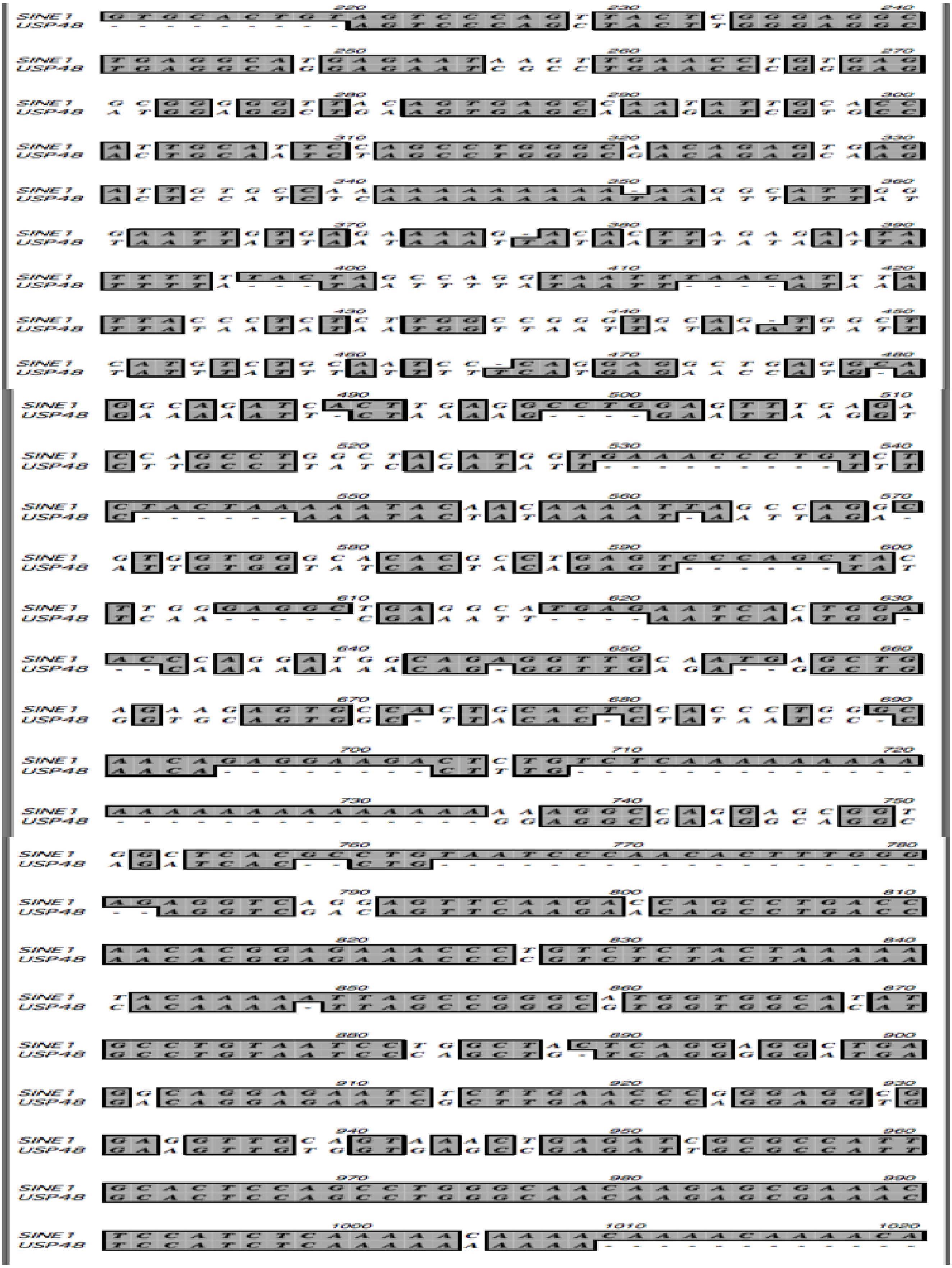

Supplement: Supplementary File 1 — Supplementary Materials (PDF, 1256 KB) [file genes-04-00226-s001.pdf]
